# Knockout of toll-like receptor impairs nerve regeneration after a crush injury

**DOI:** 10.18632/oncotarget.20206

**Published:** 2017-08-10

**Authors:** Ching-Hua Hsieh, Cheng-Shyuan Rau, Pao-Jen Kuo, Shu-Hsuan Liu, Chia-Jung Wu, Tsu-Hsiang Lu, Yi-Chan Wu, Chia-Wei Lin

**Affiliations:** ^1^ Department of Plastic and Reconstructive Surgery, Kaohsiung Chang Gung Memorial Hospital and Chang Gung University College of Medicine, Kaohsiung, Taiwan; ^2^ Center for Vascularized Composite Allotransplantation, Chang Gung Memorial Hospital, Kaohsiung, Taiwan; ^3^ Department of Neurosurgery, Kaohsiung Chang Gung Memorial Hospital and Chang Gung University College of Medicine, Kaohsiung, Taiwan; ^4^ Faculty of Health Sciences, McMaster University, Hamilton, Canada

**Keywords:** myelination, peripheral nerve regeneration, Schwann cells, sciatic nerve crush injury, toll-like receptors

## Abstract

**Background:**

Toll-like receptors (TLRs) are involved in the initiation of Schwann cell activation and subsequent recruitment of macrophages for clearance of degenerated myelin and neuronal debris after nerve injury. The present study was designed to investigate the regenerative outcome and expression of myelination-related factors in *Tlr*-knockout mice following a sciatic nerve crush injury.

**Materials and methods:**

A standard sciatic nerve crush injury, induced by applying constant pressure to the nerve with a No. 5 jeweler's forceps for 30 s, was performed in C57BL/6, *Tlr2^−/−^*, *Tlr3^−/−^*, *Tlr4^−/−^*, *Tlr5^−/−^*, and *Tlr7^−/−^* mice. Quantitative histomorphometric analysis of toluidine blue-stained nerve specimens and walking track analysis were performed to evaluate nerve regeneration outcomes. PCR Arrays were used to detect the expression of neurogenesis-related genes of dorsal root ganglia as well as of myelination-related genes of the distal nerve segments.

**Results:**

Worse nerve regeneration after nerve crush injury was found in all *Tlr-*knockout mice than in C57BL/6 mice. Delayed expression of myelin genes and a different expression pattern of myelination-related neurotrophin genes and transcription factors were found in *Tlr*-knockout mice in comparison to C57BL/6 mice. In these TLR-mediated pathways, insulin-like growth factor 2 and brain-derived neurotrophic factor, as well as early growth response 2 and N-myc downstream-regulated gene 1, were significantly decreased in the early and late stages, respectively, of nerve regeneration after a crush injury.

**Conclusions:**

Knockout of *Tlr* genes decreases the expression of myelination-related factors and impairs nerve regeneration after a sciatic nerve crush injury.

## INTRODUCTION

Upon peripheral nerve injury, Schwann cells are activated for clearance of degenerated myelin and neuronal debris by phagocytosis [1]. Rapid and efficient clearance of this debris is a prerequisite for successful nerve regeneration to proceed [2]. Within hours to days after the injury [3–6], an inflammatory reaction is elicited by Schwann cells, which leads to recruitment of macrophages, with subsequent release of many pro-inflammatory cytokines [7, 8]. In addition, both Schwann cells and macrophages can remove degenerated myelin and neuronal debris *in vitro* [9, 10] and *in vivo* [11, 12]. Delayed initiation of Schwann cell activation or macrophage recruitment would delay nerve regeneration and prolong the period of disability [8, 13].

A large family of toll-like receptors (TLRs), consisting of 10 highly homologous TLRs in humans, has been characterized [14, 15]. TLRs are transmembrane receptors and are divided into those located in the cell membrane, such as TLR1, TLR2, TLR4, TLR5, and TLR6, and those located in the membrane of the endosome, such as TLR3, TLR7, TLR8, and TLR9 [15]. Further, TLRs are pattern recognition receptors that were originally characterized in the innate immune system; they are activated by pathogen-associated molecular patterns, such as bacterial cell wall components, and endogenous TLR ligands, such as fibronectin, heparan sulfate, fragmented hyaluronic acid, high mobility group B1, hyaluronan, and heat shock proteins 60 and 70 [16–19]. Moreover, double-stranded RNA and single-stranded RNA have also been described as endogenous danger signals and could be recognized by TLR3 [20] and TLR7 [21], respectively. Upon axotomy, TLR1, TLR2, TLR3, TLR4, and TLR7 become strongly induced in the peripheral nerves and are involved in Schwann cell activation in response to the endogenous danger signals [2, 22, 23]. In addition, stimulation of Schwann cells leads to expression of TLRs [24, 25]. Lee et al. had previously demonstrated the expression of TLRs in rat Schwann cells, and showed that TLR2, TLR3, and TLR4 are highly expressed in these cells [26]. Goethals et al. have reported expression of a broad range of TLRs (TLR1–9) in Schwann cells; in particular, TLR3, TLR4, and TLR7 are highly expressed, while the expression levels were markedly lower in motor and sensory neurons [2]. In Schwann cells, the expressed TLRs have been shown to be functional [2, 25] and lead to increased expression of pro-inflammatory mediators [24, 26–28], which contribute to the Wallerian degeneration process. In *Tlr2^−/−^* and *Tlr4^−/−^* mice, reduced recruitment of macrophages resulted in persistent myelin debris in the distal nerve stump, and a significant delay in the Wallerian degeneration process was found during nerve regeneration [29].

The innate TLR response not only facilitates removal of inhibitory myelin and neuronal debris, but also upregulates local production of neurotrophins [26, 30, 31]. In addition, Schwann cell myelination is controlled by many neurotrophins, as well as by myelination-related transcription factors [32]. For example, we have previously reported that nerve regeneration is impaired in *Tlr2^−/−^* and *Tlr4^−/−^* mice with a delayed expression of the critical re-myelination transcription factors Oct6 and Sox10 [33]. However, the roles of other TLRs in nerve regeneration and expression of myelination-related transcription factors and neurotrophins are less explored.

Therefore, the present study was designed to investigate the regenerative outcome and the expression of myelination-related factors in *Tlr*-knockout mice after a sciatic nerve crush injury.

## RESULTS

### Knockout of Tlr causes poor nerve regeneration

**Figure 1:**
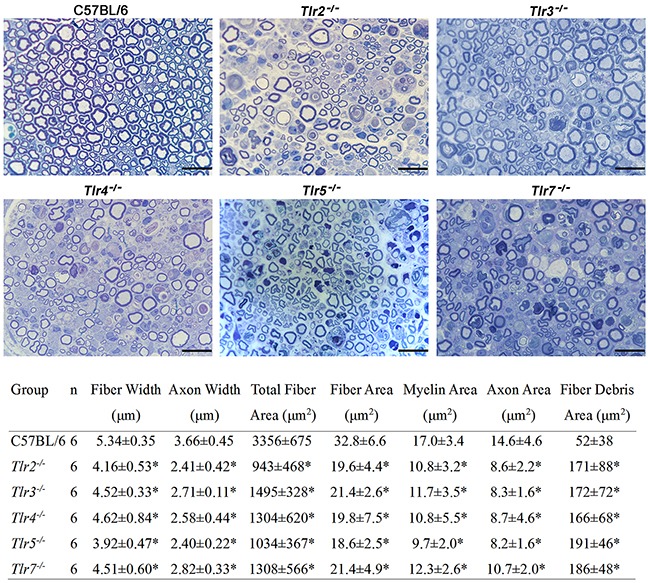
Quantitative histomorphometric analysis of the toluidine blue-stained nerve specimens 5-mm distal to the injured site in C57BL/6, *Tlr2^−/−^*, *Tlr3^−/−^*, *Tlr4^−/−^*, *Tlr5^−/−^*, and *Tlr7^−/−^* mice at post-crush day 10, with representative histological sections (× 1000) obtained distal to the nerve crush injury site on post-operative day 10, stained with toluidine blue The magnification bars represent 10 μm.

The toluidine blue-stained axial semi-thin section of the nerve, taken 5 mm distal to the injured site, in 6 mice groups (C57BL/6, *Tlr2^−/−^*, *Tlr3^−/−^*, *Tlr4^−/−^*, *Tlr5^−/−^*, and *Tlr7^−/−^*) on post-operative day 10 are illustrated in Figure 1. In the histomorphometric analysis, all the investigated *Tlr*-knockout mice presented worse nerve regeneration than the C57BL/6 mice. On day 10, the specimens of *Tlr2^−/−^*, *Tlr3^−/−^*, *Tlr4^−/−^*, *Tlr5^−/−^*, and *Tlr7^−/−^* mice presented significantly smaller fiber width, axon width, total fiber area, fiber area, myelin area, and axon area, as well as a significantly greater fiber debris area than those in the C57BL/6 mice (*n* = 6).

**Figure 2:**
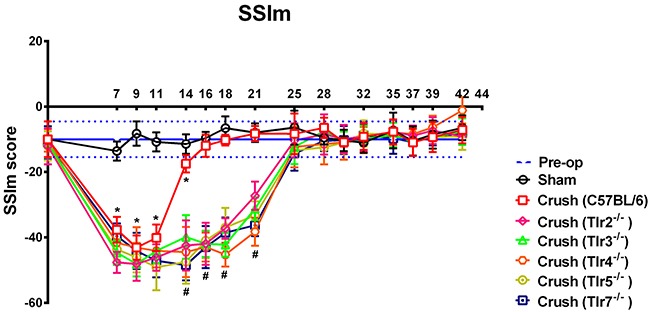
The sciatic static index in mice (SSIm) in the CatWalk gait analysis of sham-operated and experimental mice after sciatic nerve crush injury (n = 10) were recorded thrice a week for 1–7 weeks The range indicated with horizontal blue lines along the x-axis represent the preoperative values of the experimental mice prior to crush injury. * indicated p < 0.05 of C57BL/6 receiving nerve crush injury versus sham control; ^#^ indicated p < 0.05 of each *Tlr*-knockout mice versus C57BL/6 receiving nerve crush injury.

The CatWalk gait analysis (Figure 2) revealed that the values of sciatic static index in mice (SSIm) in the C57BL/6 mice were decreased the most at post-crush day 9 and then recovered by day 16. However, there were still significant decreases in SSIm in all *Tlr*-knockout mice at the corresponding time points of day 16 to 21, as compared with that of C57BL/6 mice. The SSIm values of all *Tlr*-knockout mice had recovered by post-crush day 25.

### Post-crush gene expression of distal nerve segment of C57BL/6 mice

**Figure 3:**
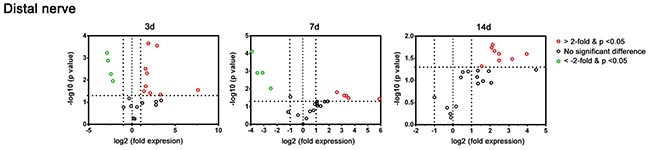
Plot of the differentially-expressed genes in a distal nerve segment of C57BL/6 mice in the PCR array at post-crush days 3, 7, and 14 Genes showing at least 2-fold differential expression and P < 0.05 between experimental and control groups were considered significant and are indicated in red (up-regulation) or green (down-regulation) colors.

As shown in Figure 3 and Table 1, at post-crush day 3, the custom PCR array showed significant (2-fold) upregulation of 9 genes (*Gdnf, Sox2, Tgfb1, Jun, Bdnf, Nrg1, Notch1, Hdac1,* and *Igf2*) in the distal nerve segment of C57BL/6 mice after a crush injury. In addition, 5 (*Gdnf, Jun, Bdnf, Sox2,* and *Tgfb1*) and 8 genes (*Gdnf, Sox2, Tgfb1, Bdnf, Pou3f2, Nrg1, Nrdg1,* and *Hdac1*) were significantly upregulated at post-crush days 7 and 14, respectively. Persistent expression, with 5-fold up-regulation throughout 14 days after the nerve crush injury, was found in 4 genes (*Gdnf, Sox2, Tgfb1, and Bdnf)*. Expression of *Jun* was observed on days 3 and 7, but not on day 14 after nerve crush injury.

**Table 1:** Differentially-expressed genes in a distal nerve segment of C57BL/6 mice in the PCR array at post-crush days 3, 7, and 14

3 days			7 days			14 days		
Genes	Fold	*p*-value	Genes	Fold	*p*-value	Genes	Fold	*p*-value
*Gdnf*	197.85	0.03	*Gdnf*	59.66	0.04	*Gdnf*	15.68	0.03
*Sox2*	9.81	0.04	*Jun*	11.46	0.03	*Sox2*	9.13	0.03
*Tgfb1*	7.41	0.001	*Bdnf*	10.25	0.02	*Tgfb1*	5.60	0.04
*Jun*	6.32	0.04	*Sox2*	9.14	0.02	*Bdnf*	5.58	0.03
*Bdnf*	5.14	0.001	*Tgfb1*	6.20	0.02	*Pou3f2*	4.70	0.02
*Nrg1*	3.33	0.01	*Plp1*	0.18	0.01	*Nrg1*	4.41	0.02
*Notch1*	3.01	0.02	*Mal*	0.12	0.001	*Nrdg1*	4.24	0.02
*Hdac1*	2.97	0.003	*Pmp22*	0.09	0.001	*Hdac1*	2.91	0.04
*Igf2*	2.61	0.03	*Mbp*	0.07	0.001			
*Plp1*	0.22	0.01						
*Pmp22*	0.19	0.02						
*Mal*	0.15	0.001						
*Mbp*	0.14	0.001						

**Figure 4:**
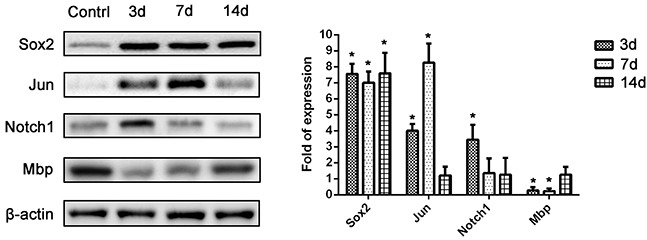
Western blot analysis of protein levels of 4 selected representative differentially-expressed genes (*Sox2*, *Jun*, *Notch1*, and *Mbp*) from the PCR array *, P < 0.05 vs. control group.

In contrast, significantly decreased expression of myelin genes (*Plp1, Pmp22, Mal,* and *Mbp*) was found at post-crush days 3 and 7, but not on day 14. Representative differentially-expressed genes (*Sox2, Jun, Notch1,* and *Mbp*) were selected for protein level detection in the distal nerve segment of C57BL/6 mice by means of western blotting (Figure 4). The Sox2 level of sciatic nerve increased significantly at post-crush day 3 and persisted till the post-crush day 14. The c-Jun increased at post-crush day 3 and 7, and returned to normal level at day 14. The expression of Notch1 increased at post-crush day 3 and returned to normal level at day 7. The Mbp level showed a significant down-regulation at post-crush day 3 to day 7, with recovery at day 14. The temporal protein expression pattern of these selected representative genes were similar to that of the transcripts identified in the PCR array.

### Post-Crush gene expression of distal nerve segment of Tlr-Knockout mice

**Figure 5:**
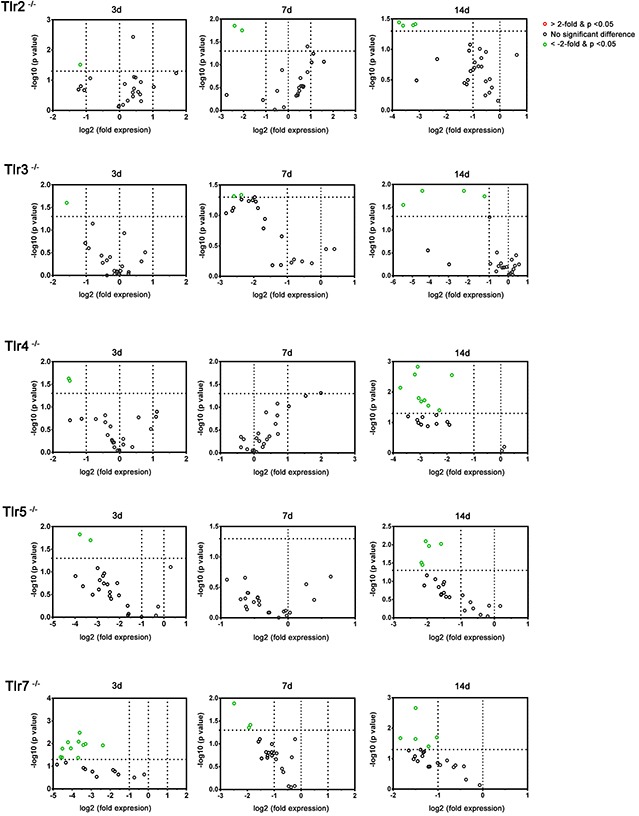
Plot of the differentially-expressed genes in distal nerve segment of *Tlr2^−/−^*, *Tlr3^−/−^*, *Tlr4^−/−^*, *Tlr5^−/−^*, and *Tlr7^−/−^* mice in the PCR array at post-crush day 3, 7, and 14 Genes showing at least 2-fold differential expression and P < 0.05 between experimental and control groups were considered significant and are indicated in red (up-regulation) or green (down-regulation) colors.

The PCR array experiments revealed that, at post-crush day 3, *Igf2* was significantly down-regulated in the *Tlr2^−/−^*, *Tlr3^−/−^*, *Tlr4^−/−^*, *Tlr5^−/−^*, and *Tlr7^−/−^* mice, while *Bdnf* was significantly down-regulated in the *Tlr4^−/−^*, *Tlr5^−/−^* and *Tlr7^−/−^* mice, as compared to the C57BL/6 mice (Figure 5 and Table 2). Moreover, down-regulation of 7 additional genes was found in the *Tlr7^−/−^* mice. On post-crush day 7, *Igf2* and *Bdnf* were significantly down-regulated in *Tlr2^−/−^*, *Tlr3^−/−^*, and *Tlr7^−/−^* mice, as compared to C57BL/6 mice. In addition, *Pou3f2* was significantly down-regulated in *Tlr7^−/−^* mice. On post-crush day 7, *Erg2* and *Ndrg1* were significantly down-regulated in the *Tlr2^−/−^*, *Tlr3^−/−^*, *Tlr4^−/−^*, *Tlr5^−/−^*, and *Tlr7^−/−^* mice, as compared to C57BL/6 mice. Additionally, 3 (*Pou3f2, Hdac1, and Sox2*) and 1 (*Nrg1*) genes were significantly down-regulated in the *Tlr4^−/−^* and *Tlr7^−/−^* mice, respectively.

**Table 2:** Differentially-expressed genes in a distal nerve segment of *Tlr2^−/−^*, *Tlr3^−/−^*, *Tlr4^−/−^*, *Tlr5^−/−^*, and *Tlr7^−/−^* mice in the PCR array than those of C57BL/6 mice at corresponding post-crush days 3, 7, and 14

*Tlr2*^−/−^			*Tlr3*^−/−^			*Tlr4*^−/−^			*Tlr5*^−/−^			*Tlr7*^−/−^		
Genes	Fold	*p*-value	Genes	Fold	*p*-value	Genes	Fold	*p*-value	Genes	Fold	*p*-value	Genes	Fold	*p*-value
*Igf2*	0.44	0.03	*Igf2*	0.33	0.03	*Igf2*	0.35	0.02	*Igf2*	0.10	0.02	*Igf2*	0.09	0.01
						*Bdnf*	0.32	0.01	*Bdnf*	0.06	0.02	*Bdnf*	0.06	0.01
												*Jun*	0.08	0.04
												*Egr2*	0.08	0.003
												*Ndrg1*	0.03	0.01
												*Pou3f2*	0.04	0.04
												*Sox10*	0.04	0.04
												*Hdac1*	0.10	0.01
												*Notch1*	0.05	0.01
												*Tgfb1*	0.19	0.01
												*Sox2*	0.06	0.02
Differentially-expressed genes of distal nerve segment of toll-like receptor knockout mice in the PCR array at post-crush day 7														
***Tlr2*^−/−^**			***Tlr3*^−/−^**			***Tlr4*^−/−^**			***Tlr5*^−/−^**			***Tlr7*^−/−^**		
**Genes**	**Fold**	***p*-value**	**Genes**	**Fold**	***p*-value**	**Genes**	**Fold**	***p*-value**	**Genes**	**Fold**	***p*-value**	**Genes**	**Fold**	***p*-value**
*Igf2*	0.22	0.03	*Igf2*	0.26	0.05							*Igf2*	0.27	0.04
*Bdnf*	0.11	0.03	*Bdnf*	0.24	0.04							*Bdnf*	0.26	0.04
												*Pou3f2*	0.17	0.01
Differentially-expressed genes of distal nerve segment of toll-like receptor knockout mice in the PCR array at post-crush day 14														
***Tlr2*^−/−^**			***Tlr3*^−/−^**			***Tlr4*^−/−^**			***Tlr5*^−/−^**			***Tlr7*^−/−^**		
**Genes**	**Fold**	***p*-value**	**Genes**	**Fold**	***p*-value**	**Genes**	**Fold**	***p*-value**	**Genes**	**Fold**	***p*-value**	**Genes**	**Fold**	***p*-value**
*Erg2*	0.08	0.03	*Erg2*	0.02	0.02	*Erg2*	0.12	0.02	*Erg2*	0.21	0.03	*Erg2*	0.43	0.04
*Ndrg1*	0.09	0.04	*Ndrg1*	0.06	0.02	*Ndrg1*	0.08	0.01	*Ndrg1*	0.22	0.03	*Ndrg1*	0.35	0.02
*Mal*	0.11	0.04	*Mal*	0.02	0.03	*Pou3f2*	0.15	0.03	*Mal*	0.33	0.01	*Nrg1*	0.28	0.02
*Pmp22*	0.11	0.04	*Pmp22*	0.42	0.02	*Hdac1*	0.11	0.03	*Pmp22*	0.26	0.01	*Mal*	0.35	0.002
						*Sox2*	0.20	0.04	*Mbp*	0.24	0.01	*Pmp22*	0.49	0.02
						*Mal*	0.28	0.002						
						*Pmp22*	0.12	0.001						
						*Mbp*	0.11	0.003						
						*Plp1*	0.14	0.02						

In the TLR-mediated pathways, 2 genes in the early stage (*Igf2* and *Bdnf*) and 2 genes (*Erg2* and *Ndrg1*) in the late stage seem to play an important role in nerve regeneration following a crush injury. Furthermore, in contrast to the significantly decreased expression of myelin genes (*Plp1, Pmp22, Mal,* and *Mbp*) in C57BL/6 mice at post-crush days 3 and 7, but not on day 14, decreased expression of various myelin genes (*Plp1, Pmp22, Mal, and Mbp*) was found at post-crush day 14 in *Tlr*-knockout mice. Compared to the C57BL/6 mice, a delay of myelin gene expression and a different expression pattern of myelination-related neurotrophin genes and transcription factors were found in *Tlr*-knockout mice after nerve injury.

### Post-Crush gene expression of DRGs of C57BL/6 mice

**Figure 6:**
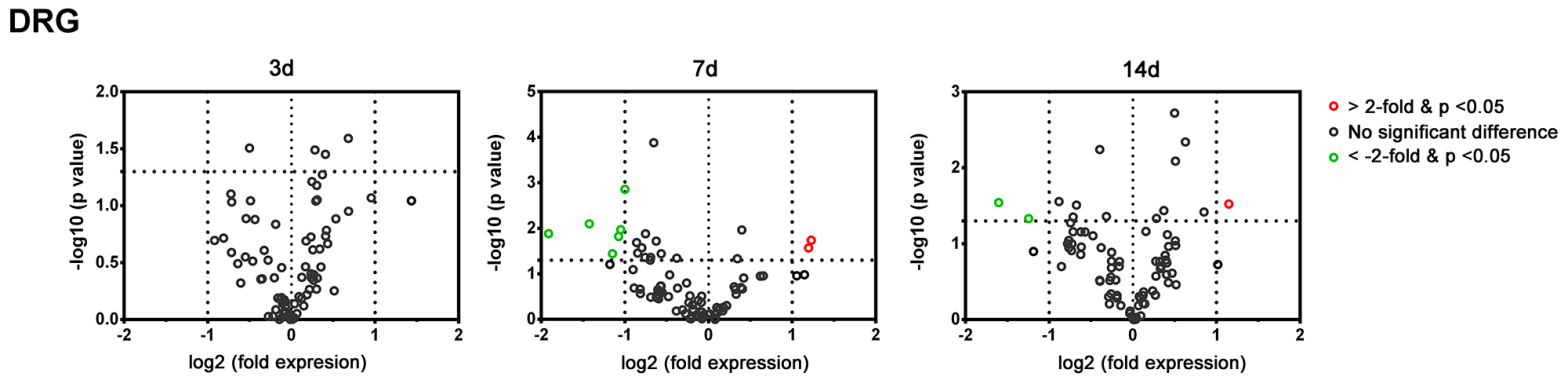
Plot of the differentially-expressed genes in L4-L6 dorsal root ganglia of C57BL/6 mice in the PCR array at post-crush day 3, 7, and 14 Genes showing at least 2-fold differential expression and P < 0.05 between experimental and sham control groups were considered significant and are indicated in red (up-regulation) or green (down-regulation) colors.

**Figure 7:**
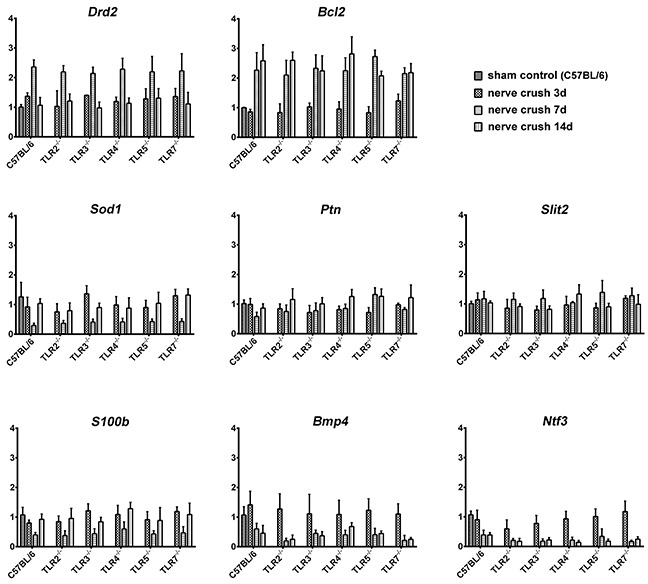
Real time quantitative PCR measurement of the expression level of those differentially-expressed genes in the DRGs of C57BL/6 and *Tlr*-knockout mice

As shown in Figure 6 and Table 3, there was no significant dysregulated genes at post-crush day 3 in the DRGs of C57BL/6 mice after a crush injury. At post-crush days 7 and 14, two genes (*Drd2* and *Bcl2*) and one gene (*Bcl2*) were significantly upregulated, respectively, and six (*Sod1, Ptn, Slits, Bmp4, S100b,* and *Ntf3*) and two genes (*Bmp4* and *Ntf3*) were significantly decreased, respectively. Using RT-qPCR to measure the expression level of dysregulated genes (Table 4) in the DRGs of *Tlr*-knockout mice following nerve crush injury, we found that the expression pattern of these genes were similar to that of the transcripts identified in the PCR array. In addition, the knockout of *Tlr2, Tlr3, Tlr4, Tlr5,* and *Tlr7* did not significantly change the expression level of these dysregulated genes in the DRGs, when compared to those of C57BL/6 mice (Figure 7).

**Table 3:** Differentially-expressed genes in L4-L6 dorsal root ganglia of C57BL/6 mice in the PCR array at post-crush days 3, 7, and 14 against C57BL/6 sham control mice

3 days			7 days			14 days		
Genes	Fold	*p*-value	Genes	Fold	*p*-value	Genes	Fold	*p*-value
			*Drd2*	2.21	0.03	*Bcl2*	2.02	0.03
			*Bcl2*	2.08	0.04	*Bmp4*	0.42	0.04
			*Sod1*	0.48	0.01	*Ntf3*	0.33	0.02
			*Ptn*	0.47	0.02			
			*Slit2*	0.45	0.03			
			*Bmp4*	0.44	0.04			
			*S100b*	0.37	0.01			
			*Ntf3*	0.27	0.01			

**Table 4:** The dysregulated genes of the DRGs identified from PCR array in C57BL/6 mice after a nerve crush injury

No.	Abbreviation	Gene ID	Description	Primer catalog number
1	*Bcl2*	12043	B-cell leukemia/lymphoma 2	PPM02918F
2	*Bmp4*	12159	bone morphogenetic protein 4	PPM02998F
3	*Drd2*	13489	dopamine receptor D2	PPM04228A
4	*Ntf3*	18205	neurotrophin 3	PPM04325A
5	*Ptn*	19242	pleiotrophin	PPM03042F
6	*S100b*	20203	S100 protein, beta polypeptide, neural	PPM04492B
7	*Slit2*	20563	slit homolog 2	PPM37706A
8	*Sod1*	20655	superoxide dismutase 1, soluble	PPM03582A

### T cells and macrophages expression in distal nerve segment

**Figure 8:**
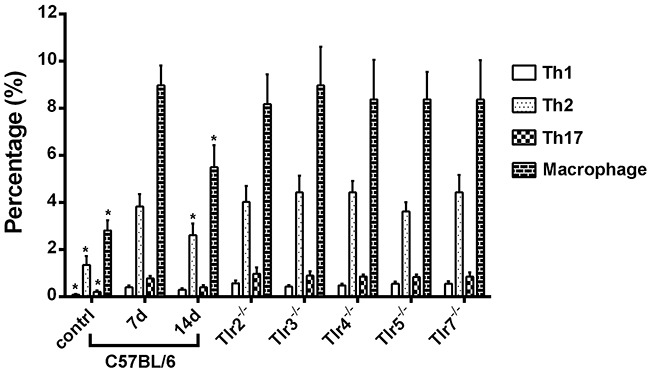
The percentages of CD4^+^IL4^+^ Th1, CD4^+^IFNγ^+^ Th2, CD4^+^IL17A^+^ Th17 cells, and CD11b^+^F4/80^+^ macrophages in the distal sciatic nerve segment of C57BL/6 mice in sham control and at post-crush days 7 and 14 as well as of *Tlr*-knockout mice at post-crush day 7 * indicated p < 0.05 versus post-crush day 7.

Using the flow cytometry to measure the abundance of T cells and macrophages in the distal nerve segment after nerve crush injury (Supplementary Figure 1), we found the percentages of residual CD4^+^IL4^+^ Th1, CD4^+^IFNγ^+^ Th2, CD4^+^IL17A^+^ Th17 cells, and CD11b^+^F4/80^+^ macrophages in the sciatic nerve segment of C57BL/6 mice account for 0.1%, 1.35%, 0.2%, and 2.8% of total cell population, respectively (Figure 8). After nerve crush injury, there were a significant increased percentage of these cells to 0.4% (Th1), 3.8% (Th2), 0.8% (Th17), and 9.0% (macrophages) of total cell population in the nerve at post-crush day 7. Then the percentage of cells was significantly decreased to 0.3% (Th1), 2.6% (Th2), 0.4% (Th17), and 5.5% (macrophages) at post-crush day 14. However, in the nerve segment of *Tlr2^−/−^*, *Tlr3^−/−^*, *Tlr4^−/−^*, *Tlr5^−/−^*, and *Tlr7^−/−^*) mice at post-crush day 7, the percentage of Th1, Th2, Th17 cells, and macrophages did not significantly differ from those measured in the C57BL/6 mice at post-crush day 7 (Figure 8). These results suggested that the population of T cells and macrophages increased in the nerve after a crush injury but the knockout of *Tlr* gene did not significantly change the accumulated population of T cells and macrophages in the nerve.

## DISCUSSION

In this study, we aimed to investigate the regenerative outcome and expression of myelination-related factors after a sciatic nerve crush injury in *Tlr*-knockout mice. We have demonstrated that nerve regeneration after sciatic nerve crush injury in *Tlr2^−/−^*, *Tlr3^−/−^*, *Tlr4^−/−^*, *Tlr5^−/−^*, and *Tlr7^−/−^* mice was worse than that in C57BL/6 mice, according to histomorphometric measurement and CatWalk gait analysis. The *Tlr*-knockout mice not only presented a significantly more prominent fiber debris area, but also a cluster of significantly down-regulated myelination-related factors, as compared to C57BL/6 mice. The various TLRs signal through different signaling pathways. While TLR2 forms heteromers with TLR1 or TLR6 to regulate intracellular signaling [34], the other TLRs form homodimers [35, 36] to initiate signaling pathways via either a myeloid differentiation 88 (MyD88)-dependent pathway or a TIR-domain-containing adapter-inducing interferon (TRIF)-dependent pathway [37]. The majority of TLRs (TLR1/2, 5, 2/6, 7, and 9) signal primarily through the MyD88 pathway; in contrast, TLR3 acts via the TRIF pathway and TLR4 uniquely signals via both MyD88 and TRIF pathways. However, in this study, the expression of down-regulated myelination-related factors was not markedly different among TLRs with distinct downstream signaling pathways or between those TLRs located in the cell membrane or in the endosomal membrane. A complex combination of endogenous TLR ligands in the degenerated nerve segment after nerve crush injury may be involved in the mechanism.

In this study, the knockout of *Tlr* gene did not significantly change the expression level of the dysregulated neurogenesis-related genes in the DRGs, indicating the impaired nerve degeneration in *Tlr*-knockout mice was attributed to condition of the distal nerve segment but not innervated neurons. In addition, the knockout of *Tlr* gene did not significantly change the accumulation of T cells and macrophages in the distal nerve segment after a crush injury, implying an more important role of Schwann cells than those immune cells here to explain the impaired nerve degeneration in *Tlr*-knockout mice. Reprogramming of gene expression patterns in Schwann cells during an injury response is regulated by the actions of distal regulatory elements that integrate the actions of multiple transcription factors [38]. In this study, persistent expression of 4 genes (*Gdnf, Sox2, Tgfb1, and Bdnf)* was found in C57BL/6 mice throughout 14 days after nerve crush injury. GDNF is a potent survival factor for many types of neurons [39–41] and is up-regulated in Schwann cells to support axonal growth after nerve injury [42, 43]. Sox2 influences transcription at the level of elongation in Schwann cells and is important for Schwann cell development [44]. Sox2 also activates ephrin-B signaling, which is required for Schwann cells to migrate in an organized fashion to allow axons to grow back properly [45]. TGFβ1 is a potent Schwann cell mitogen and controls Schwann cell division *in vivo* [32]. It is also critical for Wallerian degeneration after nerve injury, as the knockdown of TGFβ1 expression results in a reduction of Schwann cell proliferation and apoptosis [46]. c-Jun induced in Schwann cells of the nerve distal to the injury governs major aspects of Wallerian degeneration by activating a repair program and changing the phenotype of Schwann cells [47]. The absence of c-Jun results in a striking failure of functional recovery, with such Schwann cells being unable to generate the repair phenotype that is essential for regeneration [47]. Notably, c-Jun has also been reported to mediate suppression of myelin genes after nerve injury [48].

In this study, a large number of myelin genes (*Pmp22, Mal, and Mbp*) showed remarkably similar patterns of expression; it is likely that a specific set of transcription factors, including EGR2 and POU-domain transcription factors, commonly regulates these genes [49, 50]. However, in contrast to the significantly decreased expression of myelin genes (*Plp1, Pmp22, Mal,* and *Mbp*) in C57BL/6 at post-crush days 3 and 7, but not on day 14, the decreased expression of these genes were only found at post-crush day 14 in *Tlr*-knockout mice. In this study, the different expression patterns of myelination-related factors in Schwann cells of *Tlr*-knockout mice after nerve injury could not be explained solely by the delayed demyelination that was shown by a delayed expression of the myelin genes. Notably, in these TLR-mediated pathways, *Igf2* and *Bdnf,* and *Erg2* and *Ndrg1,* seem to be important in the early and late stage, respectively, of nerve regeneration after a crush injury.

Among these factors, IGF2 is a nerve- and muscle-derived soluble factor that supports neuronal survival and nerve regeneration [51] and promotes *in vitro* neurite outgrowth from sympathetic [52], sensory neurons [52], and motoneurons [53]. IGF2 can increase nerve regeneration [54], and is expressed in a spatially and temporally distinct manner within Schwann cells of the injured nerve [55].

BDNF is a neurotrophin richly expressed in denervated Schwann cells, and facilitates creation of a permissive growth environment for regenerating axons [56]. It promotes neuronal survival, maturation, and growth [57]. BDNF promotes Schwann cell migration in the distal nerve stump [58] and increases sprouting of axons from the proximal end of the cut nerve into the denervated nerve stumps [59].

NDRG1 protein is highly expressed in peripheral nerves and is localized in the cytoplasm of myelinating Schwann cells [60, 61]. In contrast, there is a lack of NDRG1 expression in sensory and motor neurons as well as in their axons [61]. After peripheral nerve axotomy, NDRG1 expression is maintained in the early stage of myelin degradation, but is then markedly depleted at the end-stage of myelin degradation [61], and finally recovers during remyelination. NDRG1 had also been suggested to be important in the terminal differentiation of Schwann cells during nerve regeneration [60].

EGR2 is a zinc finger transactivator that increases a diverse array of genes required for peripheral nerve myelination [38, 62]. EGR2 directly increases expression of desert hedgehog, which is critically involved in development, maintenance, and regeneration of myelinated fibers [62].

In this study, the down-regulation of *Igf2* and *Bdnf* in the early stage as well as *Erg2* and *Ndrg1* in the late stage in the distal nerve segment may explain the impaired nerve regeneration of *Tlr*-knockout mice after a nerve crush injury. However, although the myelination-related factors in downstream signal pathways of different TLRs are quite similar but not the same; therefore, further investigation is needed to clarify the regulated pathways of different TLRs in more details, this also comprise a limitation in this study.

In conclusion, nockout of *Tlr* genes decreases the expression of myelination-related transcription factors and neurotrophin genes and impairs nerve regeneration after a sciatic nerve crush injury. This study has yielded insights into the pathways involved in nerve generation, laying the basis for further research into treatments of nerve injuries.

## MATERIALS AND METHODS

### Animals

*Tlr2^−/−^*(B6.129-Tlr2^tm1Kir^/J), *Tlr3^−/−^* (B6N.129S1-Tlr3^tm1Flv^/J), *Tlr4^−/−^* (C57BL/10ScNJ), *Tlr5^−/−^* (B6.129S1-Tlr5^tm1Flv^/J), and *Tlr7^−/−^* (B6.129S1-Tlr7^tm1Flv^/J) mice were purchased from Jackson Laboratory (Bar Harbor, ME). C57BL/6 mice were purchased from the National Laboratory Animal Center, Taiwan. In this study, only 8-12-week-old male mice, weighing between 20 and 30 g, were used. All housing conditions were established and surgical procedures, analgesia, and assessments were performed in an AAALAC-accredited, specific pathogen-free facility, following national and institutional guidelines. Animal protocols were approved by the IACUC of Chang Gung Memorial Hospital.

### Animal surgery and sample collection

On the day of surgery (indicated as day 0), mice were anesthetized by intramuscular injection of 25 mg/kg ketamine and 50 mg/kg xylazine. The right sciatic nerve at the mid-thigh level was exposed and was then crushed with No. 5 Jeweler forceps, using consistent pressure for 30 s. After release of the forceps, a 10-0 Ethilon suture (Micro suture Ethicon, Somerville, NJ) passed through the epineurium only, without constriction, was used to mark the injured site to facilitate further harvest of the nerve specimen. The right sciatic nerve of sham-operated mice was left untouched, except for a mark made with an epineurial suture at the corresponding site. Then, all mice were allowed to awake and recover in a separate postoperative care room. In this study, totally 84 C57BL/6 mice and 34 mice of each *Tlr*-knockout species were used. Mice in 6 groups (C57BL/6, *Tlr2^−/−^*, *Tlr3^−/−^*, *Tlr4^−/−^*, *Tlr5^−/−^*, and *Tlr7^−/−^*) were re-anesthetized for harvesting the innervated L4-L6 dorsal root ganglia (DRGs) as well as one cm of the nerve distal to the injured site and were then euthanized, at post-crush days 3, 7, and 14. The samples from sham-operated C57BL/6 mice were used as a sham control. The DRGs and nerve samples were used for PCR array (n = 4 for each group at each time point). Besides, the nerve samples were harvested from additional C57BL/6 mice (sham control, at post-crush days 3, 7, and 14) were used for Western blot analysis (n = 6 at each time point) to validate the protein expression of the dysregulated genes identified from the PCR array. Additional mice (n=6 for each group) were sacrificed at day 10 and the distal nerves were harvested for histomorphometric analysis. Additional ten mice in each of seven groups (C57BL/6 sham control, nerve crush injury in C57BL/6, *Tlr2^−/−^*, *Tlr3^−/−^*, *Tlr4^−/−^*, *Tlr5^−/−^*, and *Tlr7^−/−^* mice) were used for walking track analysis. Additional C57BL/6 mice (sham control, post-crush days 7 and 14) and *Tlr*-knockout mice at post-crush day 7 were sacrificed (n=6 for each group) to measure the infiltration of immune cells into the distal nerve by flow cytometry.

### Quantitative assessment of sciatic nerve architecture

The axial 1 cm of the nerve distal to the injured site was isolated and fixed at 4°C with 3% glutaraldehyde (Polysciences Inc., Warrington, PA), washed in 0.1 M phosphate buffer (pH 7.2), post-fixed with 1% osmium tetroxide (Fisher Scientific, Pittsburgh, PA), dehydrated in graded ethanol solutions, and embedded in Araldite 502 (Polysciences Inc.). Axial semi-thin sections, with 1-μm-thick nerve specimens, taken at a 5-mm distance from the injured site, were stained with 1% toluidine blue for histomorphometric analysis. Binary image analysis was used for semi-automated quantitative analysis of multiple components of nerve histomorphometry; this was performed by an observer blinded to experimental conditions [33, 63]. Total myelinated fiber counts were measured based on 6 representative fields at 1000 × magnification. Fiber width, axon width, total fiber area, fiber area, myelin area, axon area, and fiber debris area was calculated and compared.

### CatWalk gait analysis

Before the sciatic nerve crush injury, a 2-week training was conducted for gait analysis using The CatWalk system (Noldus Information Technology, Wageningen, Netherlands), which consists of a glass runway that is lit by a white fluorescent tube. Under normal circumstances, internal reflection causes the light to be restricted to the glass surface plate. When a paw touches the glass surface, the light exits the glass, thereby only illuminating the corresponding contact areas on the glass floor. The run of the animal across the runway is detected by a video camera positioned underneath the glass plate. The signal is digitized in 50 half-frames/s, and the data are acquired, compressed, stored, and eventually analyzed by the CatWalk software program.

During this 2-week period, we employed a 12-g/day food deprivation protocol, with a reward of food pellets placed at the end of the runway in each run to motivate the animals, which were trained to make consecutive runs over a glass runway without interruptions. Importantly, gait velocity was controlled in runs with a stable runway crossing time of between 1 and 2 s in the CatWalk. After sciatic nerve crush injury, an average number of 8 replicate crossings made by each mouse in the sham-operated or experimental groups (n = 10 in each group) were recorded thrice a week for 1−7 weeks, on days 7, 9, 11, 14, 16, 18, 21, 25, 28, 32, 35, 37, 39, and 42. The CatWalk software was used to analyze crossings that had at least 5 cycles of complete steps.

### Gene expression in RT^2^ profiler PCR arrays

Total RNA was isolated with the use of an RNeasy Mini kit (Qiagen, Valencia, CA) under the implementation of the RNase-Free DNase digestion step according to the manufacturer's instruction. An Agilent 2100 BioAnalyzer was used to confirm the RNA integrity. An RT^2^ First Strand kit was used for reverse transcription. To detect the expressed genes of the distal nerve segment, RT^2^ SYBR Green/ROX qPCR Master Mix was used for quantitative PCR on a custom Qiagen RT^2^ Profiler PCR Array of mouse (Qiagen), which included 4 myelin genes, as well as 16 myelination-related transcription factors or neurotrophin genes (Table 5). To detect the expressed genes of the L4-L6 DRGs, the Mouse Neurogenesis RT^2^; Profiler™ PCR Array (Qiagen), which profiles the expression of 84 genes (Supplementary Table 1) related to related to the regulation of key neurogenesis processes such as the cell cycle and cell proliferation, differentiation, motility, and migration. The 7500 Real-Time PCR System (Applied Biosystems, Carlsbad, CA) was used to amplify the cDNA. The expression level of target genes was calculated according to Qiagen RT^2^ Profiler PCR Array handbook, with a panel of housekeeping genes, including beta-actin (*Actb*) and glyceraldehyde-3-phosphate dehydrogenase (*Gapdh*), which were used as reference genes. The genes included in the RT^2^ Profiler PCR Array whose threshold cycle fell above the 35^th^ cycle were excluded from data presentation. Quality control was confirmed with the positive PCR controls and reverse transcription controls of the PCR array, and only the results that passed quality checks in PCR array reproducibility and the lack of genomic DNA contamination were included. Genes had to show at least 2-fold differential expression and P < 0.05 between experimental and control groups to be considered significant in this study.

**Table 5:** The myelin genes and myelination-related transcription factors or neurotrophin genes included in the customized Qiagen RT^2^ Profiler PCR Mouse Array

No.	Abbreviation	Gene ID	Ensemble	Description
Myelin				
1	*Mal*	4118	ENSMUSG00000027375	myelin and lymphocyte protein, T cell differentiation protein
2	*Mbp*	17196	ENSMUSG00000041607	myelin basic protein
3	*Plp*	18823	ENSMUSG00000031425	proteolipid protein 1
4	*Pmp22*	18858	ENSMUSG00000018217	peripheral myelin protein 22
Myelination-related neurotrophic and transcript factors				
1	*Bdnf*	12064	ENSMUSG00000048482	brain derived neurotrophic factor
2	*Pou3f2 (Brn2)*	18992	ENSMUSG00000095139	POU domain, class 3, transcription factor 2
3	*Gdnf*	14573	ENSMUSG00000022144	glial cell line derived neurotrophic factor
4	*Igf2*	16000	ENSMUSG00000048583	insulin-like growth factor 2
5	*Egr2 (Krox20)*	13654	ENSMUSG00000037868	early growth response 2
6	*Nrg1*	211323	ENSMUSG00000062991	neuregulin 1
7	*Pou3f1 (Oct6)*	18991	ENSMUSG00000090125	POU domain, class 3, transcription factor 1
8	*Sox10*	20665	ENSMUSG00000033006	SRY (sex determining region Y)-box 10
9	*Hdac1*	433759	ENSMUSG00000028800	histone deacetylase 1
10	*Ndrg1*	10397	ENSG00000104419	N-myc downstream regulated gene 1
11	*Jun*	16476	ENSMUSG00000052684	jun proto-oncogene
12	*Notch1*	18128	ENSMUSG00000026923	notch 1
13	*Ntf3*	18205	ENSMUSG00000049107	neurotrophin 3
14	*Pax3*	18050	ENSMUSG00000004872	paired box 3
15	*Sox2*	20674	ENSMUSG00000074637	SRY (sex determining region Y)-box 2
16	*Tgfb1*	21803	ENSMUSG00000002603	transforming growth factor, beta 1

### Western blot analysis

Western blotting was used to confirm the protein level of those differentially-expressed genes of distal nerve segment that were identified from the PCR array of those C57BL/6 mice after nerve crush injury. The distal nerve specimens of 6 C57BL/6 mice specific for post-crush days 3, 7, and 14 were harvested and homogenized with tissue protein extraction reagent T-PER^TM^ (Pierce, Waltham, MA) containing phosphatase and protease inhibitors. The protein samples in 30 μg were resolved on 10% SDS-polyacrylamide gels and transferred to polyvinylidenedifluoride membranes. Blots were blocked with 5% skim milk in Tween-20/ phosphate-buffered saline, and incubated with various primary antibodies, i.e., rabbit anti–Sox2 (Millipore Biotechnology, Billerica, MA), anti–c–Jun (Millipore Biotechnology), anti-Notch1 (Novus Biologicals, Littleton, CO), anti-Mbp (Novus Biologicals), and anti-β-actin (Millipore Biotechnology), at 4°C overnight. The blots were then incubated with horseradish peroxidase-conjugated secondary antibodies at room temperature for 60 min, and developed with ECL™ Western Blotting Systems (Amersham Pharmacia Biotech, Little Chalfont, UK). The protein bands were detected using a FluorChem 8900 imaging system and quantified with the AlphaEaseFC software (Alpha Innotech Corp, Santa Clara, CA).

### Real time quantitative PCR

Real time quantitative PCR (RT-qPCR) was used to validate the expression level of those differentially-expressed genes, that were identified from the PCR array, in the DRGs of C57BL/6 mice and to measure their expression level in the *Tlr*-knockout mice. An RNeasy Mini kit (Qiagen) was used to isolate total RNA from the DRGs of C57BL/6 at indicated groups (sham control, post-crush days 3, 7, and 14) and *Tlr*-knockout (*Tlr2^−/−^*, *Tlr3^−/−^*, *Tlr4^−/−^*, *Tlr5^−/−^*, and *Tlr7^−/−^*) mice at post-crush days 3, 7, and 14. A High-Capacity cDNA Reverse Transcription (ABI 4368814, Applied Biosystems, Foster City, CA) was used for reverse transcription. This converted DNA was subjected to RT-qPCR with specific primer designed for the PCR array by Qiagen using an Applied Biosystems® 7500 Real-Time PCR Systems (Applied Biosystems) in a 96-well optical plate format. Amplification was carried out in 25 μl volume reactions containing Power SYBR Green PCR Master Mix (ABI 4367659, Applied Biosystems) according to the manufacturer's recommendations. The cycling conditions consisted of a 30-minute reverse transcription step at 50°C, a 2 min denaturation step at 95°C and 40 amplification cycles of 15 sec at 95°C and 1 min at 60°C. Fluorescence was acquired during each extension step and reactions were performed in triplicate. With PCR-grade water as the negative controls and endogenous reference (*Gapdh*) for normalization, the target mRNA levels were obtained relativized to the sham-operated samples using the formula 2^−ΔΔCt^.

### Flow cytometry analysis

One cm distal nerve segment was removed from the C57BL/6 mice in sham control and at post-crush days 7 and 14 as well as from *Tlr*-knockout mice at post-crush day 7 (n = 6 for each group at each time point). The cells isolated from the distal nerve segments of each subgroup were analyzed with flow cytometry to calculate the population of T cells (n=3) and macrophages (n=3). A single-cell suspension was created by grinding the nerve segment with The gentleMACS™ Octo Dissociator (Miltenyi Biotec, Taiwan), then passing cells through a 30 μm MACS^®^ SmartStrainers (Miltenyi Biotec) and the cells were collected in Petri dishes. The cells were fixed and permeabilized with Fixation/Permeabilization Buffers (BD Biosceinces) and then washed with PBS according to the manufacturer's instructions. The isolated cells were stained with a mouse Th1/Th2/Th17 phenotyping kit (BD Biosceinces) with the fluorescent antibodies: peridinin-chlorophyll-protein complex - CY5.5 (PerCP-Cy5.5)-conjugated anti-CD4 as well as allophycocyanin (APC)-conjugated IL4, fluorescein isothiocyanate (FITC)-conjugated IFNγ, and phycoerythrin (PE)-conjugated IL17A for detecting Th1, Th2 or Th17, or stained with FITC-conjugated anti-CD11b Ab or PE-conjugated anti-F4/80 Ab (BD Biosceinces) for macrophages. Flow cytometry was performed on a BD LSR II flow cytometer (BD Biosciences) with CellQuest Pro software (BD Biosciences).

### Statistical Analysis

All the results were presented as mean ± standard error. An overall analysis of the differences between group means was calculated by one-way analysis of variance (ANOVA). A post-hoc Fisher's least significant difference test was used to compare the differences between groups. In all cases, statistical significant was set at P < 0.05.

## SUPPLEMENTARY MATERIALS FIGURES AND TABLES





## Abbreviations

BDNF: brain derived neurotrophic factor
ERG2: early growth response 2
IGF2: insulin-like growth factor 2
MAL: myelin and lymphocyte protein, T cell differentiation protein
MBP: myelin basic protein
NDRG1: N-myc downstream regulated gene 1
PLP: proteolipid protein 1
PMP22: peripheral myelin protein 22
SSIm: sciatic static index in mice
TGFB1: transforming growth factor, beta 1
TLRs: toll-like receptors
