# Supplementary material for: Knockout of toll-like receptor impairs nerve regeneration after a crush injury
Source: Oncotarget. 2017 Aug 10;8(46):80741–56. doi: 10.18632/oncotarget.20206 (PMC5655236; doi:10.18632/oncotarget.20206)
Supplement: Supplementary file 1 [file oncotarget-08-80741-s001.pdf]

Knockout of toll-like receptor impairs nerve regeneration after a crush injury

SUPPLEMENTARY MATERIALS

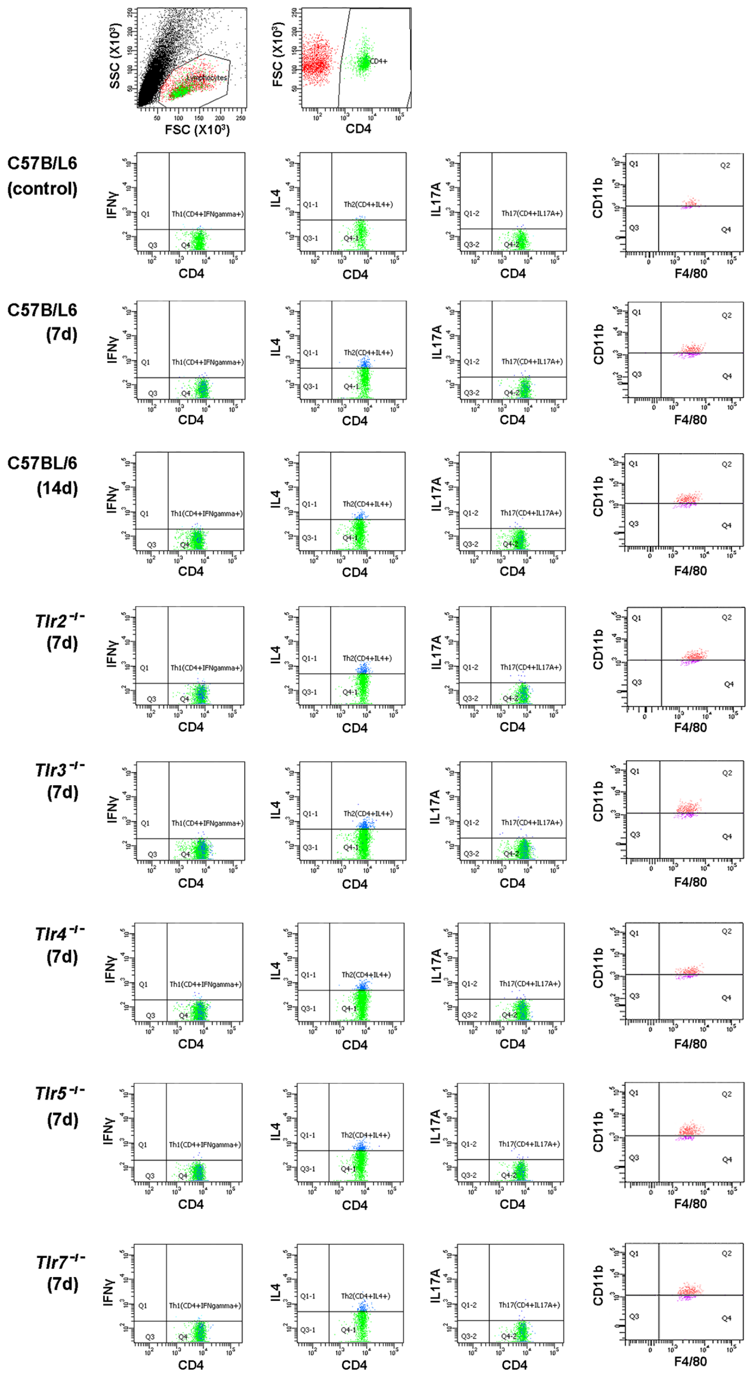

Supplementary Figure 1: Flow cytometry analysis of immunoreactive T cells and macrophages in triplicate of the distal nerve segment after a crush injury.

Supplementary Table 1: Gene table of Mouse Neurogenesis RT² Profiler™ PCR Array.

See Supplementary File 1
