# Supplementary material for: Knockout of toll-like receptor impairs nerve regeneration after a crush injury
Source: Oncotarget. 2017 Aug 10;8(46):80741–56. doi: 10.18632/oncotarget.20206 (PMC5655236; doi:10.18632/oncotarget.20206)
Supplement: Supplementary file 2 [file oncotarget-08-80741-s002.docx]

**Supplementary Table 1: Gene table of Mouse Neurogenesis RT² Profiler™ PCR Array**

| **Position** | **Unigene** | **GeneBank** | **Symbol** | **Description** |
| --- | --- | --- | --- | --- |
| A01 | Mm.255464 | NM_009599 | Ache | Acetylcholinesterase |
| A02 | Mm.298908 | NM_001008533 | Adora1 | Adenosine A1 receptor |
| A03 | Mm.333734 | NM_009630 | Adora2a | Adenosine A2a receptor |
| A04 | Mm.311854 | NM_007439 | Alk | Anaplastic lymphoma kinase |
| A05 | Mm.38469 | NM_009685 | Apbb1 | Amyloid beta (A4) precursor protein-binding, family B, member 1 |
| A06 | Mm.305152 | NM_009696 | Apoe | Apolipoprotein E |
| A07 | Mm.277585 | NM_007471 | App | Amyloid beta (A4) precursor protein |
| A08 | Mm.56897 | NM_009711 | Artn | Artemin |
| A09 | Mm.136217 | NM_008553 | Ascl1 | Achaete-scute complex homolog 1 (Drosophila) |
| A10 | Mm.257460 | NM_009741 | Bcl2 | B-cell leukemia/lymphoma 2 |
| A11 | Mm.1442 | NM_007540 | Bdnf | Brain derived neurotrophic factor |
| A12 | Mm.103205 | NM_007553 | Bmp2 | Bone morphogenetic protein 2 |
| B01 | Mm.6813 | NM_007554 | Bmp4 | Bone morphogenetic protein 4 |
| B02 | Mm.439764 | NM_007559 | Bmp8b | Bone morphogenetic protein 8b |
| B03 | Mm.142275 | NM_009871 | Cdk5r1 | Cyclin-dependent kinase 5, regulatory subunit 1 (p35) |
| B04 | Mm.370777 | NM_145990 | Cdk5rap2 | CDK5 regulatory subunit associated protein 2 |
| B05 | Mm.448632 | NM_203491 | Chrm2 | Cholinergic receptor, muscarinic 2, cardiac |
| B06 | Mm.422634 | NM_133828 | Creb1 | CAMP responsive element binding protein 1 |
| B07 | Mm.21013 | NM_008176 | Cxcl1 | Chemokine (C-X-C motif) ligand 1 |
| B08 | Mm.12871 | NM_010025 | Dcx | Doublecortin |
| B09 | Mm.27256 | NM_007864 | Dlg4 | Discs, large homolog 4 (Drosophila) |
| B10 | Mm.4875 | NM_007865 | Dll1 | Delta-like 1 (Drosophila) |
| B11 | Mm.41970 | NM_010077 | Drd2 | Dopamine receptor D2 |
| B12 | Mm.247259 | NM_007889 | Dvl3 | Dishevelled 3, dsh homolog (Drosophila) |
| C01 | Mm.3374 | NM_010110 | Efnb1 | Ephrin B1 |
| C02 | Mm.252481 | NM_010113 | Egf | Epidermal growth factor |
| C03 | Mm.258397 | NM_177821 | Ep300 | E1A binding protein p300 |
| C04 | Mm.290822 | NM_001003817 | Erbb2 | V-erb-b2 erythroblastic leukemia viral oncogene homolog 2, neuro/glioblastoma derived oncogene homolog (avian) |
| C05 | Mm.473689 | NM_008006 | Fgf2 | Fibroblast growth factor 2 |
| C06 | Mm.295533 | NM_010227 | Flna | Filamin, alpha |
| C07 | Mm.4679 | NM_010275 | Gdnf | Glial cell line derived neurotrophic factor |
| C08 | Mm.589 | NM_008155 | Gpi1 | Glucose phosphate isomerase 1 |
| C09 | Mm.278672 | NM_008169 | Grin1 | Glutamate receptor, ionotropic, NMDA1 (zeta 1) |
| C10 | Mm.318567 | NM_207225 | Hdac4 | Histone deacetylase 4 |
| C11 | Mm.390859 | NM_008235 | Hes1 | Hairy and enhancer of split 1 (Drosophila) |
| C12 | Mm.29581 | NM_010423 | Hey1 | Hairy/enhancer-of-split related with YRPW motif 1 |
| D01 | Mm.103573 | NM_013904 | Hey2 | Hairy/enhancer-of-split related with YRPW motif 2 |
| D02 | Mm.103615 | NM_013905 | Heyl | Hairy/enhancer-of-split related with YRPW motif-like |
| D03 | Mm.983 | NM_010556 | Il3 | Interleukin 3 |
| D04 | Mm.906 | NM_010784 | Mdk | Midkine |
| D05 | Mm.24001 | NM_025282 | Mef2c | Myocyte enhancer factor 2C |
| D06 | Mm.2389 | NM_001081049 | Kmt2a | Myeloid/lymphoid or mixed-lineage leukemia 1 |
| D07 | Mm.436793 | NM_001039934 | Map2 | Microtubule-associated protein 2 |
| D08 | Mm.400253 | NM_010882 | Ndn | Necdin |
| D09 | Mm.5014 | NM_010883 | Ndp | Norrie disease (pseudoglioma) (human) |
| D10 | Mm.4636 | NM_010894 | Neurod1 | Neurogenic differentiation 1 |
| D11 | Mm.266665 | NM_010896 | Neurog1 | Neurogenin 1 |
| D12 | Mm.42017 | NM_009718 | Neurog2 | Neurogenin 2 |
| E01 | Mm.255596 | NM_010897 | Nf1 | Neurofibromatosis 1 |
| E02 | Mm.135266 | NM_008711 | Nog | Noggin |
| E03 | Mm.290610 | NM_008714 | Notch1 | Notch gene homolog 1 (Drosophila) |
| E04 | Mm.485843 | NM_010928 | Notch2 | Notch gene homolog 2 (Drosophila) |
| E05 | Mm.103641 | NM_013708 | Nr2e3 | Nuclear receptor subfamily 2, group E, member 3 |
| E06 | Mm.208439 | NM_176930 | Nrcam | Neuron-glia-CAM-related cell adhesion molecule |
| E07 | Mm.153432 | NM_178591 | Nrg1 | Neuregulin 1 |
| E08 | Mm.271745 | NM_008737 | Nrp1 | Neuropilin 1 |
| E09 | Mm.266341 | NM_010939 | Nrp2 | Neuropilin 2 |
| E10 | Mm.267570 | NM_008742 | Ntf3 | Neurotrophin 3 |
| E11 | Mm.39095 | NM_008744 | Ntn1 | Netrin 1 |
| E12 | Mm.327698 | NM_011855 | Tenm1 | Odd Oz/ten-m homolog 1 (Drosophila) |
| F01 | Mm.37289 | NM_016967 | Olig2 | Oligodendrocyte transcription factor 2 |
| F02 | Mm.397111 | NM_013625 | Pafah1b1 | Platelet-activating factor acetylhydrolase, isoform 1b, subunit 1 |
| F03 | Mm.299254 | NM_033620 | Pard3 | Par-3 (partitioning defective 3) homolog (C. elegans) |
| F04 | Mm.1371 | NM_008781 | Pax3 | Paired box gene 3 |
| F05 | Mm.439659 | NM_008782 | Pax5 | Paired box gene 5 |
| F06 | Mm.487124 | NM_013627 | Pax6 | Paired box gene 6 |
| F07 | Mm.440553 | NM_008900 | Pou3f3 | POU domain, class 3, transcription factor 3 |
| F08 | Mm.246550 | NM_011143 | Pou4f1 | POU domain, class 4, transcription factor 1 |
| F09 | Mm.279690 | NM_008973 | Ptn | Pleiotrophin |
| F10 | Mm.292510 | NM_009007 | Rac1 | RAS-related C3 botulinum substrate 1 |
| F11 | Mm.310772 | NM_019413 | Robo1 | Roundabout homolog 1 (Drosophila) |
| F12 | Mm.192580 | NM_194053 | Rtn4 | Reticulon 4 |
| G01 | Mm.100144 | NM_011313 | S100a6 | S100 calcium binding protein A6 (calcyclin) |
| G02 | Mm.235998 | NM_009115 | S100b | S100 protein, beta polypeptide, neural |
| G03 | Mm.57202 | NM_009170 | Shh | Sonic hedgehog |
| G04 | Mm.482843 | NM_178804 | Slit2 | Slit homolog 2 (Drosophila) |
| G05 | Mm.276325 | NM_011434 | Sod1 | Superoxide dismutase 1, soluble |
| G06 | Mm.65396 | NM_011443 | Sox2 | SRY-box containing gene 2 |
| G07 | Mm.35784 | NM_009237 | Sox3 | SRY-box containing gene 3 |
| G08 | Mm.473190 | NM_011486 | Stat3 | Signal transducer and activator of transcription 3 |
| G09 | Mm.248380 | NM_011577 | Tgfb1 | Transforming growth factor, beta 1 |
| G10 | Mm.1292 | NM_009377 | Th | Tyrosine hydroxylase |
| G11 | Mm.44701 | NM_022312 | Tnr | Tenascin R |
| G12 | Mm.282184 | NM_009505 | Vegfa | Vascular endothelial growth factor A |
| H01 | Mm.328431 | NM_007393 | Actb | Actin, beta |
| H02 | Mm.163 | NM_009735 | B2m | Beta-2 microglobulin |
| H03 | Mm.309092 | NM_008084 | Gapdh | Glyceraldehyde-3-phosphate dehydrogenase |
| H04 | Mm.3317 | NM_010368 | Gusb | Glucuronidase, beta |
| H05 | Mm.2180 | NM_008302 | Hsp90ab1 | Heat shock protein 90 alpha (cytosolic), class B member 1 |
| H06 | N/A | SA_00106 | MGDC | Mouse Genomic DNA Contamination |
| H07 | N/A | SA_00104 | RTC | Reverse Transcription Control |
| H08 | N/A | SA_00104 | RTC | Reverse Transcription Control |
| H09 | N/A | SA_00104 | RTC | Reverse Transcription Control |
| H10 | N/A | SA_00103 | PPC | Positive PCR Control |
| H11 | N/A | SA_00103 | PPC | Positive PCR Control |
| H12 | N/A | SA_00103 | PPC | Positive PCR Control |
